# Control region sequences indicate that multiple externae represent multiple infections by *Sacculina carcini* (Cirripedia: Rhizocephala)

**DOI:** 10.1002/ece3.1177

**Published:** 2014-07-30

**Authors:** David Rees, Henrik Glenner

**Affiliations:** 1 Department of Biology, Marine Biodiversity, University of BergenThormøhlensgate 53a, Box 7800, Bergen, N-5020, Norway; 2 Center for Macroecology, Evolution and Climate, University of CopenhagenUniversitetsparken 15, 2100 Copenhagen, Denmark

**Keywords:** *Carcinus maenas*, control region, mitochondrial DNA, parasitism, population genetics, rhizocephala

## Abstract

The rhizocephalan barnacle, *Sacculina carcini*, is a common parasite of the European shore crab, *Carcinus maenas*, in which it causes significant detrimental physical and behavioral modifications. In the vast majority of cases, the external portion of the parasite is present in the form of a single sac-like externa; in rare cases, double or even triple externae may occur on the same individual host. Here, we use a highly variable DNA marker, the mitochondrial control region (CR), to investigate whether multiple externae in *S. carcini* represent infection by multiple parasites or asexual cloning developed by a single parasite individual. Sequences for multiple externae from *C. maenas* hosts from the Danish inlet, Limfjorden, and from the mud flates at Roscoff, France, were compared. In almost all cases, double or triple externae from an individual host yielded different haplotypes. In the few cases where identical haplotypes were identified from externae on a multiple-infected host, this always represented the most commonly found haplotype in the population. This indicates that in *Sacculina carcini,* the presence of multiple externae on a single host reflects infection by different individual parasites. A haplotype network of CR sequences also suggests a degree of geographical partitioning, with no shared haplotypes between the Limfjorden and Roscoff. Our data represent the first complete CR sequences for a rhizocephalan, and a unique gene order was also revealed. Although the utility of CR sequences for population-level work must be investigated further, the CR has proved a simple to use and highly variable marker for studies of *S. carcini* and can easily be applied to a variety of studies in this important parasite.

## Introduction

**Figure 1:**
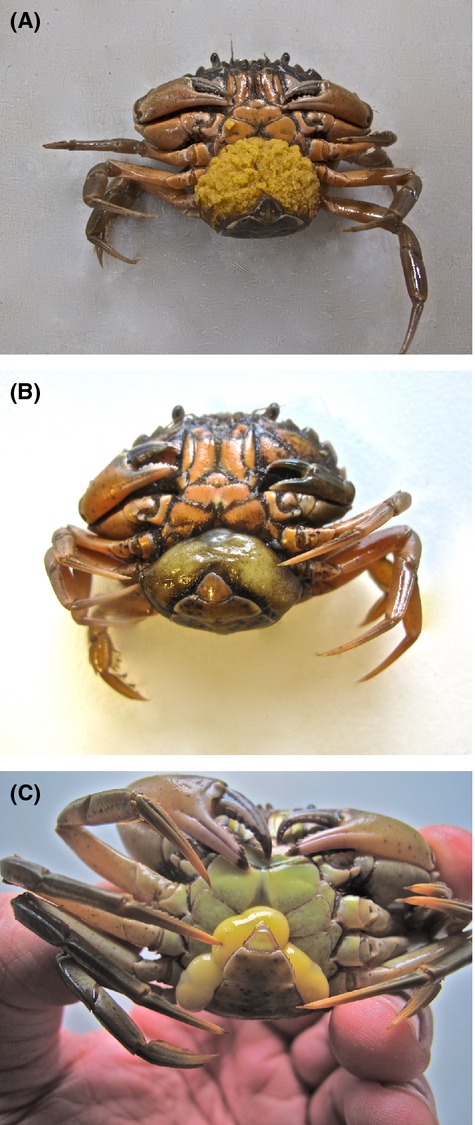
Comparison of a gravid female crab carrying its egg mass under the abdomen and mimicking rhizocephalan parasites situated at the same position. (A) Gravid female *Carcinus maenas*; (B) female *C. maenas* with a single mature *S. carcini* parasite; (C) male *C. maenas* with three recently emerged *Sacculina carcini* externae. The abdomen has been considerable broadened (feminized) as effect of the presence of the parasites.

The rhizocephalan barnacle, *Sacculina carcini*, is a parasitic castrator of the European shore crab, *Carcinus maenas*. The adult parasite consists of an external sac-like structure located at the ventral side of the host abdomen at a position where adult female crabs carry their eggs (see Fig. 1A, B). This sac, the externa, contains the reproductive organs of the parasite and communicates with an internal root-like structure, the interna, via a stalk that penetrates the abdominal cuticle of the host. The interna is an extensive structure that infiltrates most of the larger blood sinuses of the crab and serves as a trophic organ extracting nutrients from the crab hemolymph (Glenner 2001; for further details of the development of *Sacculina carcini* and its morphological and behavioral impacts on *Carcinus maenas* see, e.g., Kristensen et al. 2012; Høeg 1995; Høeg and Lutzen 1995).

Occasionally, more than one externa occurs on the same crab (Rainbow et al. 1979). This can be interpreted as either a double infection, where two individual parasite specimens are competing for the same food resource entailing that each externa is feeding from two independent internas (see Fig. 1C). Alternatively, the phenomenon can be regarded as asexual budding of two externae connected to, and feeding from, the same interna. Which of the two alternatives is in play is of crucial importance in order to understand the biology of the parasite and the host–parasite interaction. Due to the delicacy of the interna, which consists of a dense network of extremely fine root extensions, it is impossible to distinguish morphologically whether two externae are connected to a common interna (asexual externa budding), or to separate internas (independent specimens). As part of a 4-years, large-scale study on the population dynamics and biology of the European shore crab, *Carcinus maenas*, in the Danish inlet Limfjorden (see http://www.Carcinus.com), specimens with the rare occurrence of multiple externae were collected. The large quantity of crabs examined during the project allowed collection of an unprecedented number of crabs with multiple externae. This allowed a comprehensive examination of whether the observed multiple externae were caused by a single infection, multiple infections, or a combination of both alternatives. Multiple externae are documented as being developed by clonal, or asexual reproduction, in one sacculinid genus, *Polyascus*, where multiple externae on the same host is the rule (Glenner et al. 2003). In other sacculinid genera, the presence of multiple externae on the same host is rare, but notable exceptions do exist, as in *Heterosaccus dolfusi*, which parasitizes the swimming crab, *Charybdis longicollis*, a Lessepsian invader of the Mediterranean sea (Galil and Lutzen 1995). In this parasite–host system, parasitized crabs predominantly have more than one externa and 3–5 per host is common. With an extraordinary prevalence of 60–90%, the chances of a cypris larva finding a host already occupied by another parasites are much higher than finding an un-parasitized host, and it is therefore believed that the intensity observed in most cases is due to multiple infections – not asexual reproduction (Glenner and Hebsgaard 2006). However, since morphological inspections of the internal parasite is unable to distinguish the presence of more than one externa, the question of asexual budding or multiple infection can only be addressed by developing, and employing, high-resolution molecular markers.

Mitochondrial (mt) DNA is a popular marker for phylogenetic, phylogeographic, and population genetic studies at a wide range of taxonomic and geographical scales. Notable benefits of mtDNA markers include relative ease of amplification, presence of variable regions flanked by conserved stretches suitable for primer design, and predominantly nonrecombinant inheritance. The mtDNA control region (CR) is a noncoding portion of the mt genome, responsible for replication and transcription, and is usually the fastest evolving mtDNA region in invertebrates (Avise 2000; Billington 2003). The CR exhibits an evolutionary rate threefold to fivefold higher than other regions of the mt genome (Brown et al. 1993), making it a popular marker for genetic studies involving a wide range of taxa. This marker has been widely utilized in vertebrates and insects but also been applied to studies of genetic variability and population structure in a number of commercially important marine crustaceans, for example, the swimming crab *Portunus trituberculatus* (Guo et al. 2012), the scalloped lobster *Panulirus homarus* (Farhadi et al. 2013), and the mantis shrimp *Oratosquilla oratoria* (Lui et al. 2010).

Use of CR sequences in published studies of Cirripedia is few, and its use seems to be limited mainly to population and taxonomic studies involving a small number of Thoracican barnacles; *Chthamalus stellatus* (Sasson et al. 2012), *Semibalanus balanoides* (Flight et al. 2012), *Catomerus polymerus* (York et al. 2008), and *Tetraclita* spp. (Chan et al. 2007; Tsang et al. 2007; Dawson et al. 2010). No studies involving Rhizocephala, and no complete control region sequences for this group, have been published to date. The high variability associated with the CR made this an ideal candidate for our work involving *Sacculina carcini*, both for assessing population – and individual-level variation. An initial test of control region sequence data for *Sacculina carcini*, aimed at assessing suitability for population studies, indicated surprisingly high levels of variability. This presented the opportunity to investigate another aspect of *S. carcini* biology: whether (rare) double and triple infections (indicated by multiple externae) are the result of emergence by multiple individuals of *S. carcini*, or whether multiple externae can emerge from a single parasite. In this study, we present new primers and a simple method for targeting the CR in *Sacculina carcini* and examine DNA sequence variation in multiple externa from two disjunct populations.

## Methods

### Sample collection and DNA extraction

*Sacculina carcini* externa were collected from the shore crab/green crab *Carcinus maenas* from Limfjorden, Denmark, and from near Roscoff, north-west France, and preserved in 96% ethanol prior to DNA extraction. Genomic DNA extraction was performed for a total of 57 externa comprising six single infections, 21 double, and three triple infections (double and triple infections being characterized by two or three externa from a single host specimen. Five double and three single externae were collected from *C. maenas* near Roscoff and the remainder (three triple, 16 double, and three single externae) came from Limfjorden (see the data accessibility section, Table 1 for sample details). Approximately 1 mm^3^ of mantle tissue was carefully excised from individual *Sacculina carcini* externae for DNA extraction. Alternatively, eggs or nauplii larvae from the mantle cavity of the female externae were DNA extracted. Tissue from the receptacle region were carefully omitted to avoid DNA contamination from a dwarf male. All extractions were carried out using the Qiagen DNeasy Blood and Tissue kit (QIAGEN Inc., Valencia, CA, USA) or a GeneMole extraction robot, following the manufacturers’ standard protocols. Multiple parallel extractions were also performed for individual externa, along with PCR amplification and sequencing, to confirm reproducibility and specificity of DNA sequence data.

**Table 1:** *Sacculina carcini* host, location, and haplotype information. Individual hosts are numbered and double or triple externa denoted by A/B/C. Associated haplotypes indicated for each externa and GenBank accession numbers are listed for each unique haplotype. The three most common haplotypes are also indicated, as in Fig. 2, by *α*, *β*, and *γ*

Externa	Host No.	Location	Haplotype	GenBank accession
SA01	1	Roscoff	R01	KF649275
SA02	2	Roscoff	R02	KF649276
SA03	3	Roscoff	R03	KF649277
SA04	4	Limfjorden	L08 *β*	KF649263
SA05	5	Limfjorden	L17 *γ*	KF649272
SA06	6	Limfjorden	L18	KF649273
TC01A	7	Limfjorden	L17 *γ*	
TC01B		Limfjorden	L09	KF649264
TC02A	8	Limfjorden	L16	KF649271
TC02B		Limfjorden	L11 *α*	KF649266
TC03A	9	Limfjorden	L11 *α*	
TC03B		Limfjorden	L13	KF649268
TC04A	10	Limfjorden	L06	KF649261
TC04B		Limfjorden	L01	KF649256
TC05A	11	Limfjorden	L19	KF649274
TC05B		Limfjorden	L08 *β*	
TC06A	12	Limfjorden	L11 *α*	
TC06B		Limfjorden	L08 *β*	
502A	13	Roscoff	R04	KF649278
502B		Roscoff	R05	KF649279
503A	14	Roscoff	R06	KF649280
503B		Roscoff	R07	KF649281
504A	15	Roscoff	R08	KF649282
504B		Roscoff	R09	KF649283
512A	16	Roscoff	R10	KF649284
512B		Roscoff	R11	KF649285
513A	17	Roscoff	R12	KF649286
513B		Roscoff	R13	KF649287
SAC11A	18	Limfjorden	L05	KF649260
SAC12B		Limfjorden	L17 *γ*	
SAC13C		Limfjorden	L11 *α*	
SAC14A	19	Limfjorden	L14	KF649269
SAC15B		Limfjorden	L11 *α*	
SAC16C		Limfjorden	L03	KF649258
SAC17A	20	Limfjorden	L17 *γ*	
SAC18B		Limfjorden	L04	KF649259
SAC19C		Limfjorden	L15	KF649270
SAC20A	21	Limfjorden	L01	
SAC21B		Limfjorden	L08 *β*	
SAC22A	22	Limfjorden	L17 *γ*	
SAC23B		Limfjorden	L08 *β*	
SAC24A	23	Limfjorden	L02	KF649257
SAC25B		Limfjorden	L08 *β*	
SAC26A	24	Limfjorden	L10	KF649265
SAC27B		Limfjorden	L08 *β*	
SAC28A	25	Limfjorden	L17 *γ*	
SAC29B		Limfjorden	L17 *γ*	
SAC30A	26	Limfjorden	L07	KF649262
SAC31B		Limfjorden	L09	
SAC32A	27	Limfjorden	L17 *γ*	
SAC33B		Limfjorden	L08 *β*	
SAC34A	28	Limfjorden	L11 *α*	
SAC35B		Limfjorden	L17 *γ*	
SAC36A	29	Limfjorden	L12	KF649267
SAC37B		Limfjorden	L17 *γ*	
SAC38A	30	Limfjorden	L17 *γ*	
SAC39B		Limfjorden	L17 *γ*	

### Primer design and PCR

Specific polymerase chain reaction (PCR) primers were designed from flanking regions using published *S. carcini* sequences; the forward primer was designed from the single 12S rRNA sequence available in GenBank (AY520690), and the reverse primer was designed from an alignment of multiple GenBank cytochrome c oxidase 1 gene (COI) sequences. Primer design was performed using the Primer3Plus web interface (Untergasser et al. (2007); http://www.bioinformatics.nl/cgi-bin/primer3plus/primer3plus.cgi) and the following primers were selected: 12SF_Sacc (5′-TGAATTCAGATTAGGTGCAAAGA-3′) and COIR_Sacc (5′-CCCCCACTAAACCTGATCATA-3′). PCR amplifications were carried out in 25 *μ*L volumes containing 1× PCR buffer, 1.2-*μ*L 2-mmol/L dNTPs, 0.4 *μ*mol/L of each primer, 0.75 units of Takara polymerase, and 1 *μ*L of template (and ddH2O up to 25 *μ*L). PCRs were performed on a Bio-Rad C1000 Thermal with the following cycling profile: initial denaturation at 94°C for 5 min, then 35 cycles of 94°C for 30 sec, annealing at 54°C for 30 sec, and extension at 72°C for 2 min, followed by a final 72°C extension for 7 min. Amplification products were visualized on 1.5% agarose gels to confirm fragment size and quality. PCR purification was carried out by the addition of 1 unit each of exonuclease I and shrimp alkaline phosphatase (plus 0.9 *μ*L ddH2O) to 8 *μ*L of each reaction, with reactions subsequently heated to 37°C for 30 min and then 85°C for 15 min. In a small number of cases, PCR products were gel-purified using the Qiagen MinElute gel extraction kit, with the target band excised directly from the gel prior to sequencing.

### Sequencing and data analysis

Both strands of all PCR products were sequenced on an ABI 3730 capillary sequencer using the BigDye v3.1 cycle sequencing kit (Applied Biosystems, Inc., Norwalk, CT, USA) and the same primers as in the initial PCR. Forward and reverse sequences were aligned and edited in Sequencher v.5.0.1 (Gene Codes) and a contig of all sequences exported to eBioX v.1.5.1 (http://www.ebioinformatics.org) for final alignment using MUSCLE (Edgar 2004). A haplotype network was subsequently estimated using the TCS program (v.1.21; Clement et al. 2000) using the default 95% connection limit. In addition, annotation of the sequenced region was accomplished by analyses of the full mitochondrial genome for *S. carcini* (unpubl. data Podsiadlowski, L., Hecht, J., Rees D., Noever, D., Glenner, H.) using the MITOS web server (Bernt et al. 2012; http://mitos.bioinf.uni-leipzig.de/).

## Results and Discussion

### Sequence characteristics

The final aligned dataset, comprising DNA sequence data for 57 individual externa, was 812 base-pair (bp) long. The presence of insertions and deletions (indels) resulted in unaligned individual sequence lengths ranging from 795 to 803 bp. BLAST searches confirmed partial matches to *S. carcini* 12S and COI genes (http://blast.ncbi.nlm.nih.gov/Blast.cgi) and subsequent alignment of the sequenced region with the complete mitochondrial genome sequence for *S. carcini* (unpublished data) confirmed the relative positions of the flanking regions and the presence of the putative control region. Annotation of the sequenced region using the MITOS package indicated that the sequenced region comprised approximately 175 bp of 12S and 35 bp of COI, flanking a 600-bp segment of noncoding A+T-rich sequence (84% A+T). Analyses also indicated that the noncoding region between the 12S and COI genes was split by one 56-bp tRNA (tRNA Cysteine; gca), resulting in a 145 bp of noncoding sequence adjacent to 12S and 400 bp adjacent to COI. The overall length of the amplified fragment was significantly shorter than expected (see below) but with primers located in conserved, coding flanking regions, we minimized potential problems with amplification (or nondetection) of nuclear mitochondrial copies (Numts) or CR paralogs (see, e.g., Walther et al. 2011). Comparison of our coding sequence data indicates complete congruence with those held in GenBank, as well as complete agreement for our total sequence reads with an independently generated full mitochondrial *S. carcini* genome sequence (unpublished data). Analysis of this full mtDNA genome sequence identified all 13 protein coding genes, two rRNA genes, and 22 tRNA genes.

With our primers located in the 12S and CO1 genes, we had expected to amplify the NADH dehydrogenase subunit 2 (ND2) gene along with the control region, since the gene order for all Cirripedia for which data is available (with one exception, see below), as well as for the majority of crustaceans, is 12S, CR, ND2, and CO1. This also applies to ancestral pancrustaceans and arthropods (Lavrov et al. 2004). However, our sequence data indicated that for *Sacculina carcini*, ND2 was not present in this position (and the fragment was therefore ∼1 kb shorter than expected).

A survey of full mitochondrial genome sequences held in GenBank for crustaceans (18 August 2013) indicated that the majority of taxa present the expected order for genes flanking the control region (12S, CR, ND2, COI). This gene order exists in 49 of 78 noncirriped crustaceans in which the position of the control region has been annotated (an additional 23 full crustacean mitochondrial genomes have no control region specified). Different flanking genes are reported for 27 taxa (including ten species in a single amphipod genus), but all Cirripedia for which data are available conform to the majority pattern, with the exception of the single rhizocephalan barnacle for which a near-complete mitochondrial genome has been published: *Polyascus gregaria* (Yan et al. 2012). Although Yan et al. were unable to present full sequence for the control region in *P. gregaria* due to problematic repetitive elements, the gene order presented by the authors is consistent with other cirripeds except for the additional presence of ND1 between the control region and ND2.

As such, the flanking gene order for *Sacculina carcini* is, at present, unique among Cirripedia and also Crustacea. Although a predominant flanking gene order was evident from the survey of crustacean full mitochondrial genome sequences, the position of the control region appears variable in some taxa and if a few cases, two control regions have been annotated. Position and percentage A+T content support the assumption that the region sequenced for this study is likely to be the control region. However, there is a possibility that *Sacculina carcini* also possesses a second control region, since annotation of the full mitochondrial sequence for *S. carcini* (unpublished data) indicates an A+T-rich (85%) 655-bp noncoding region between the cytochrome b (cob) and 12S genes.

### Haplotype diversity

**Figure 2:**
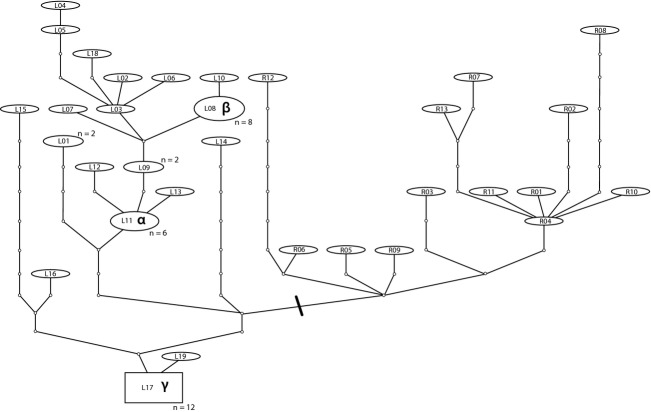
Statistical parsimony network inferred from mtDNA sequence data, with haplotypes coded “R” for Roscoff or “L” for Limfjorden. Ovals represent sampled haplotypes, branches connecting haplotypes represent nucleotide substitution steps, and small circles represent missing haplotypes. The square represents the haplotype (L17/C) inferred by TCS to be ancestral. Oval sizes are relative to haplotype frequency; details of individuals represented, along with single, double, or triple externa status and GenBank accession numbers, are presented in Table 1. The bold bar indicates the division between parts of the network corresponding to Limfjorden and Roscoff samples.

The initial group of *S. carcini* externa examined included three single externa from two locations; Roscoff, north-west France, and Limfjorden in Denmark. These six individual externa yielded six haplotypes, with 0.4–0.6% pairwise divergence among the Roscoff samples (mean = 0.5%) and 0.1–1.1% among those from Limfjorden (mean = 0.8%). Together with sequences from double or triple externa, the final dataset of 57 externa yielded 32 haplotypes, shown as a TCS haplotype network in Fig. 2. Representatives of all haplotypes shown in the TCS network have been deposited in GenBank under the accession numbers KF649256–KF649287. All 13 externa from Roscoff and one-third of those from Limfjorden gave unique haplotypes. Within the Limfjorden sample, three haplotypes were found to be more common; one haplotype was shared by six individual externa, one by eight and one by 12 samples (denoted by in the TCS network (Fig. 2) by A, B, and C, respectively). The most common Limfjorden haplotype (C) contained sequences from two pairs of externa (i.e., two double infections with identical haplotypes). Although these could be viewed as possibly resulting from asexual budding in single *S. carcini* individuals, the fact that distinct haplotypes were found in all other double and triple externa suggest that these are more likely to be the result of a shared, relatively common haplotype in the Limfjorden population. Pairwise divergences among the 57 externa ranged from 0% (the two pairs of externa sharing the common Limfjorden “C” haplotype) to 1.5%, and overall pairwise divergence was 0.7%.

The absence of shared haplotypes among the initial set of single externa samples from Roscoff and Limfjorden populations was also consistent in the full dataset. No haplotypes were shared by individuals from these two locations and on examination of the haplotype network (Fig. 2) suggested that a degree of geographic structuring was present in the data. All samples (single and double infections) from Roscoff form one part of the network, distinct from the Limfjorden samples (Fig. 2). The most similar haplotypes from the two populations are still separated by five substitutions and the mean pairwise distance between externa from Limfjorden and Roscoff is 0.9%. Although preliminary, this is in contrast to the findings of Sasson et al. (2012) who reported no phylogeographic pattern among CR sequences from populations of the thoracican barnacle *Chthamalus stellatus*. Sasson et al. (2012) also reported a higher proportion of singleton haplotypes (68%) among sequenced individuals than we observed in *S. carcini* (47%).

Unfortunately, detailed geographical information regarding specific sites of individual samples was not available for the material used in this study, but this will be addressed in forthcoming work. Large-scale sampling of *S. carcini*, involving multiple localities including Roscoff and Limfjorden, is underway and material will be analyzed both with microsatellites and sequencing of the control region. This will allow us to assess congruence between the two datasets and to properly determine what degree of geographic resolution can be achieved from analyses of control region sequences.

Our work with the mitochondrial control region in *Sacculina carcini* has shown this A+T-rich region, flanked by the 12S rRNA and COI genes, to be highly variable. The level of variability observed makes this marker suitable for population-level studies and has also demonstrated utility in studies of genetic differentiation at finer scales, as in the case of multiple externa. Combined with specific primers and ease of amplification, the control region offers a great deal of promise as a marker that bridges the gap between phylogenetics and population genetics and is a welcome additional tool to studies of *S. carcini*.

### Life history conclusions extracted from the study

Observations of multiple externae are extraordinarily rare in crab populations infested by *Sacculina carcini*. This is true even in crab populations with high prevalence of the parasite, as in this study. The dataset is based on the examination of 24878 crabs collected between May 2011 and April 2012 in the Danish inlet, Limfjorden. On average, 9% of these crabs were visibly infested by *Sacculina carcini* due to the presence of externae. Of these 2239 (9%), 40 (1.79%) were double infected, and of those two (5%) were triple infected (female crabs were slightly but significantly more parasitized than male crabs). The small number of multiple-infected crabs is dramatically lower than would be expected if cypris infection on parasitized and un-parasitized crabs were indiscriminate and random. This is in accordance with the study of (Rainbow et al. 1979).

Considering the rarity of multiple *S. carcini* externae on infected hosts, it is worth speculating as to possible mechanisms that might be involved in the observed pattern, that is, why multiple infections are so rare. The relative rarity of multiple externa may be linked to a possible preference of infecting cypris larvae for uninfected crabs, with chemical cues involved in signaling infection status. In other barnacles, attraction of conspecifics via chemical and other cues has been documented (see, e.g., Clare et al. 1994; Dreanno et al. 2007) but signaling of infection status by *S. carcini* remains uninvestigated. Active avoidance of infected hosts in favor of searching for an uninfected crab may be unlikely due to the small size (250 *μ*m) and limited energy resources (5 days; Glenner et al. 1989; Glenner and Werner 1998) of cypris larvae. However, if two (or in even rarer cases, three) cypris larvae successfully infect a new host at the same time, then this could occasionally lead to simultaneous establishment and later emergence of multiple externae.

Alternatively, rarity of multiple externae might be explained by the fact that once a virgin externa has emerged through the abdominal cuticle of the host, host molting is arrested and competing parasites are unable to emerge (Høeg 1995; Høeg and Lutzen 1995). The result of this “first past the post” scenario would be that although multiple cypris larvae might initially infect a host, external emergence by one parasite would prevent others from utilizing the hosts’ resources for developing their reproductive apparatus. Again, in rare cases, two or more parasites might simultaneously emerge, giving rise to multiple externae.

### Future perspectives

The most widely used methods of genotyping compromises restriction-fragment-length polymorphism (RFLP), random-amplified polymorphism detection (RAPD), amplified-fragment-length polymorphism (AFLP), microsatellite genotyping, and single-nucleotide polymorphisms (SNP). For nonmodel species, with no previously existing procedure protocols, the time and money spent for developing and optimizing the methods to a new species are often disproportionally high compared to the outcome. Our study demonstrates that DNA sequence data from the highly variable mitochondrial control region is an inexpensive, easy, and robust alternative method to genetically differentiate specimens of the parasitic barnacles, *Sacculina carcini*. This marker also possesses broad utility; as well as testing for the presence/absence of asexual reproduction in adult parasites, we have successfully used *Sacculina*-specific CR amplification to screen for endoparasitic internas in potentially infected hosts without visible externae, and we will be further testing the resolution of CR sequences for small-scale population studies. Ease of use, along with the level of variation observed in *S. carcini*, makes the application of this marker for genotyping studies in other rhizocephalan species an attractive and exciting proposition.

## Data Accessibility

Table 1 Location and haplotype details for sequenced externa. Samples are grouped together in rows indicating single, double (A/B), or triple (A/B/C) externa present on a single *C. maenas* host. Haplotype codes are the same as those used in Fig. 2; GenBank accession numbers are indicated for each of the 32 unique haplotypes.

## References

[b1] Avise JC (2000). Phylogeography: the history and formation of species.

[b2] Bernt M, Donath A, Jühling F, Externbrink F, Florentz C, Fritzsch G (2012). MITOS: Improved de novo Metazoan Mitochondrial Genome Annotation. Mol. Phylogenet. Evol.

[b3] Billington N, Hallerman EM (2003). Mitochondrial DNA. Population genetics: principles and applications for Fisheries Scientists.

[b4] Brown JR, Beckenbach T, Smith MJ (1993). Intraspecific DNA sequence variation of the mitochondrial control region of white sturgeon (*Acipenser transmontanus*. Mol. Biol. Evol.

[b5] Chan BKK, Tsang LM, Ma KY, Hsu C-H, Chu KH (2007). Taxonomic revision of the acorn barnacles *Tetraclita japonica* and *Tetraclita formosana* (Crustacea: Cirripedia) in East Asia based on a combined molecular and morphological analysis. Bull. Mar. Sci.

[b6] Clare AS, Freet RK, McClary M (1994). On the antennular secretion of the cyprid of *Balanus amphitrite*, and its role as a settlement pheromone. J. Mar. Biol. Assoc. U.K.

[b7] Clement M, Posada D, Crandall KA (2000). TCS: a computer program to estimate gene genealogies. Mol. Ecol.

[b8] Dawson MN, Grosberg RK, Stuart YE, Sanford E (2010). Population genetic analysis of a recent range expansion: mechanisms regulating the poleward range limit in the volcano barnacle. Mol. Ecol.

[b9] Dreanno C, Kirby RR, Clare AS (2007). Involvement of the barnacle settlement-inducing protein complex (SIPC) in species recognition at settlement. J. Exp. Mar. Biol. Ecol.

[b10] Edgar RC (2004). MUSCLE: multiple sequence alignment with high accuracy and high throughput. Nucleic Acids Res.

[b11] Farhadi A, Farhamand H, Nematollahi MA, Jeffs A, Lavery SD (2013). Mitochondrial DNA population structure of the scalloped lobster *Panulirus homarus* (Linnaeus 1758) from the West Indian Ocean. ICES J. Mar. Sci.

[b12] Flight PA, O'Brien MA, Schmidt PS, Rand DM (2012). Genetic structure and the North American postglacial expansion of the barnacle, *Semibalanus balanoides*. J. Hered.

[b13] Galil B, Lutzen J (1995). Biological observations on *Heterosaccus dollfusi* Boschma (Cirripedia, Rhizocephala), a parasite of *Charybdis longicollis* Leene (Decapoda, Brachyura), a lessepsian migrant to the Mediterranean. J. Crustac. Biol.

[b14] Glenner H (2001). Cypris metamorphosis, injection and earliest internal development of the Rhizocephalan *Loxothylacus panopaei* (Gissler). Crustacea: Cirripedia: Rhizocephala: Sacculinidae. J. Morphol.

[b15] Glenner H, Hebsgaard M (2006). Phylogeny and evolution of life history strategies of the Parasitic Barnacles (Crustacea, Cirripedia, Rhizcephala). Mol. Phylogenet. Evol.

[b16] Glenner H, Werner M (1998). Increased susceptibility of recently moulted *Carcinus maenas* (L.) to attack by the parasitic barnacle *Sacculina carcini* Thompson 1836. J. Exp. Mar. Biol. Ecol.

[b17] Glenner H, Hoeg J, Klysner A, Larsen B (1989). Cypris ultrastructure, metamorphosis and sex in 7 families of parasitic barnacles (Crustacea, Cirripedia, Rhizocephala). Acta Zoologica.

[b18] Glenner H, Lutzen J, Takahashi T (2003). Molecular and morphological evidence for a monophyletic clade of asexually reproducing Rhizocephala: Polyascus, new genus (*Cirripedia*. J. Crustac. Biol.

[b19] Guo E, Liu Y, Cui Z, Li X, Cheng Y, Wu X (2012). Genetic variation and population structure of swimming crab (*Portunus trituberculatus*) inferred from mitochondrial control region. Mol. Biol. Rep.

[b20] Høeg JT (1995). The biology and life-cycle of the Rhizocephala (Cirripedia). J. Mar. Biol. Assoc. U.K.

[b21] Høeg JT, Lutzen J (1995). Life cycle and reproduction in the Cirripedia Rhizocephala. Oceanogr. Mar. Biol.

[b22] Kristensen T, Nielsen AI, Jørgensen AI, Mouritsen KN, Glenner H, Christensen JT (2012). The selective advantage of host feminization: a case study of the green crab *Carcinus maenas* and the parasitic barnacle *Sacculina carcini*. Mar. Biol.

[b23] Lavrov D, Brown WM, Boore JL (2004). Phylogenetic position of the Pentastomida and (pan)crustacean relationships. Proc. R. Soc. Lond. B.

[b24] Lui KKY, Leung PTY, Ng WC, Leung KMY (2010). Genetic variation of *Oratosquilla oratoria* (Crustacea: Stomatopoda) across Hong Kong waters elucidated by mitochondrial control region sequences. J. Mar. Biol. Assoc. U.K.

[b25] Rainbow P, Ford M, Hepplewhite I (1979). Absence of gregarious settling behavior by female Cypris larvae of British parasitic Rhizocephalan Barnacles. J. Mar. Biol. Assoc. U. K.

[b26] Sasson N, Simon-Blecher N, Achituv Y (2012). New molecular markers for revealing the population structure of *Chthamalus stellatus* in the Mediterranean and the eastern Atlantic. MEPS.

[b27] Tsang LM, Chan BKK, Ma KY, Hsu CS, Chu KH (2007). Lack of mtDNA and morphological differentiation between two acorn barnacles *Tetraclita japonica* and *T. formosana* differing in parietes colours and geographical distribution. Mar. Biol.

[b28] Untergasser A, Nuijveen H, Rao X, Bisseling T, Geurts R, Leunissen JAM (2007). Primer3Plus, an enhanced web interface to Primer3. Nucleic Acids Res.

[b29] Walther E, Schöfl G, Mrotzek G, Haryanti, Sugama K, Saluz HP (2011). Paralogous mitochondrial control region in the giant tiger shrimp, *Penaeus monodon* (F.) affects population genetics inference: a cautionary tale. Mol. Phylogenet. Evol.

[b30] Yan J, Zhou J, Li P, Sun H, Zhou K (2012). Nearly complete mitochondrial genome of *Polyascus gregaria* and the phylogenetic relationships among maxillopodans. Mol. Biol. Rep.

[b31] York KL, Blacket MJ, Appleton BR (2008). The Bassian Isthmus and the major ocean currents of south-east Australia influence the phylogeography and population structure of a southern Australian intertidal barnacle, *Catomerus polymerus* (Darwin). Mol. Ecol.

